# Coagulation Factor Xa Induces Proinflammatory Responses in Cardiac Fibroblasts via Activation of Protease-Activated Receptor-1

**DOI:** 10.3390/cells10112958

**Published:** 2021-10-30

**Authors:** Elisa D’Alessandro, Billy Scaf, Chantal Munts, Arne van Hunnik, Christopher J. Trevelyan, Sander Verheule, Henri M. H. Spronk, Neil A. Turner, Hugo ten Cate, Ulrich Schotten, Frans A. van Nieuwenhoven

**Affiliations:** 1 Departments of Biochemistry and Internal Medicine, Cardiovascular Research Institute Maastricht, Maastricht University Medical Center, 6220 MD Maastricht, The Netherlands; e.dalessandro@maastrichtuniversity.nl (E.D.); henri.spronk@maastrichtuniversity.nl (H.M.H.S.); h.tencate@maastrichtuniversity.nl (H.t.C.); 2 Department of Physiology, Cardiovascular Research Institute Maastricht, Maastricht University Medical Center, 6200 MD Maastricht, The Netherlands; b.scaf@maastrichtuniversity.nl (B.S.); c.munts@maastrichtuniversity.nl (C.M.); a.vanhunnik@maastrichtuniversity.nl (A.v.H.); s.verheule@maastrichtuniversity.nl (S.V.); schotten@maastrichtuniversity.nl (U.S.); 3 Discovery and Translational Science Department, Leeds Institute of Cardiovascular and Metabolic Medicine, School of Medicine, University of Leeds, Leeds LS2 9JT, UK; bs16cjt@leeds.ac.uk (C.J.T.); n.a.turner@leeds.ac.uk (N.A.T.); 4 Multidisciplinary Cardiovascular Research Centre, University of Leeds, Leeds LS2 9JT, UK; 5 Center for Thrombosis and Haemostasis, Gutenberg University Medical Centre, 55131 Mainz, Germany

**Keywords:** coagulation FXa, cardiac fibroblasts, inflammation, PARs

## Abstract

Coagulation factor (F) Xa induces proinflammatory responses through activation of protease-activated receptors (PARs). However, the effect of FXa on cardiac fibroblasts (CFs) and the contribution of PARs in FXa-induced cellular signalling in CF has not been fully characterised. To answer these questions, human and rat CFs were incubated with FXa (or TRAP-14, PAR-1 agonist). Gene expression of pro-fibrotic and proinflammatory markers was determined by qRT-PCR after 4 and 24 h. Gene silencing of *F2R* (PAR-1) and *F2RL1* (PAR-2) was achieved using siRNA. MCP-1 protein levels were measured by ELISA of FXa-conditioned media at 24 h. Cell proliferation was assessed after 24 h of incubation with FXa ± SCH79797 (PAR-1 antagonist). In rat CFs, FXa induced upregulation of *Ccl2* (MCP-1; >30-fold at 4 h in atrial and ventricular CF) and *Il6* (IL-6; ±7-fold at 4 h in ventricular CF). Increased MCP-1 protein levels were detected in FXa-conditioned media at 24 h. In human CF, FXa upregulated the gene expression of *CCL2* (>3-fold) and *IL6* (>4-fold) at 4 h. Silencing of *F2R* (PAR-1 gene), but not *F2RL1* (PAR-2 gene), downregulated this effect. Selective activation of PAR-1 by TRAP-14 increased *CCL2* and *IL6* gene expression; this was prevented by *F2R* (PAR-1 gene) knockdown. Moreover, SCH79797 decreased FXa-induced proliferation after 24 h. In conclusion, our study shows that FXa induces overexpression of proinflammatory genes in human CFs via PAR-1, which was found to be the most abundant PARs isoform in this cell type.

## 1. Introduction

Cardiac fibroblasts (CFs) are essential to maintain homeostasis of the heart [1]. Upon different types of cardiac injury (e.g., myocardial infarction, hypertensive heart disease), quiescent CFs acquire a more proliferative, migratory, and secretory phenotype, and can differentiate into myofibroblasts that express contractile proteins, such as alpha-smooth muscle actin (α-SMA), and increase collagen deposition [2,3,4].

Moreover, CFs have been reported to play an important role in the inflammatory processes that occur within the heart upon acute and chronic injury [5]. In the acute phase after myocardial infarction (MI), CFs respond to proinflammatory stimuli (e.g., reactive oxygen species (ROS), interleukin (IL)-1, and tumour necrosis factor alpha (TNF-α)) in the ischemic microenvironment by altering their gene expression profile and acting as inflammatory supporter cells [6]. In this phase, CFs produce large amounts of cytokines, such as IL-6, IL-1, and chemokines, such as IL-8 and monocyte chemoattractant protein (MCP)-1, supporting and modulating the recruitment of leukocytes that clear the tissue of dead cells and matrix debris [5,6].

Recently, coagulation factors, such as thrombin and factor (F) Xa, have been implicated in the activation of CFs and promotion of cardiac remodelling [7,8]. These non-haemostatic effects are mediated by activation of a family of G protein-coupled receptors, the protease-activated receptors (PARs) [9]. The PAR family comprises four different isoforms (PAR-1 to -4) variously expressed by different cell types including platelets, endothelial cells, mononuclear cells, and CFs. Serine proteases, such as thrombin and FXa, proteolytically cleave PARs at their N-terminus, thereby generating a new tethered ligand, which self-activates the receptor [10].

PAR-1 activation by thrombin promotes the expression of pro-fibrotic markers, such as α-SMA and transforming growth factor beta 1 (TGF-β1), but also of proinflammatory markers, such as IL-6 and MCP-1, in adult rat CFs [7].

Similarly, FXa has been reported to be a strong in vitro inducer of proinflammatory responses acting mainly via activation of PAR-2 [11]. In fact, PAR-2 activation has often been associated with pathological processes characterized by an increased inflammatory state (e.g., atherosclerosis) [12]. Recently, however, Friebel and colleagues reported that reduced expression of PAR-2 in endomyocardial biopsies of patients with heart failure with preserved ejection fraction (HFpEF) aggravated lymphocyte and macrophage infiltration and myocardial fibrosis [12,13]. Moreover, Shinozawa et al. showed that FXa upregulated the expression of the proinflammatory MCP-1 in human umbilical vein endothelial cells and that this effect was markedly inhibited by a selective PAR-1 inhibitor [14].

These apparently contrasting findings suggest that PAR-1 activation may also play a role in mediating FXa proinflammatory activity. Moreover, the specific contribution of PAR-1 and PAR-2 activation on FXa signalling on CF has not been fully characterised yet. Therefore, the aim of this study was to investigate the effect of FXa on adult rat and human CFs and clarify the role of PAR-mediated proinflammatory signalling in these cells.

## 2. Materials and Methods

### 2.1. Isolation and Culture of Adult Rat CF

Adult surplus rats (Lewis or Wistar strains, aged between 5 and 52 weeks) were euthanized according to the guidelines of Animals Act 1986 (Scientific Procedures).

The hearts were excised, and atrial and ventricular tissue was separately minced into small pieces and digested with 2 mg/mL of Worthington Collagenase Type II (Merck KGaA, Darmstadt, Germany) as previously described [15,16]. Both atrial and ventricular CFs were isolated using differential plating and cultured until 80% confluency in Dulbecco’s Modified Eagle’s medium (DMEM; no. 22320, Gibco, Invitrogen, Breda, the Netherlands) supplemented with 10% foetal bovine serum (FBS, Gibco) and gentamicin (50 µg/mL, Gibco). Cells were harvested, frozen and stored in liquid nitrogen, and used from passage 1–3.

Experiments were performed with the approval of the Animal Ethical Committee of Maastricht University (DEC-2007-116, 31 July 2007) and conform to the national legislation for the protection of animals used for scientific purposes.

### 2.2. Isolation and Culture of Human Atrial CFs

All atrial biopsies were obtained from patients undergoing different cardiac surgery procedures, after informed patient consent and approval of the Institutional Ethics Committee of the Maastricht University (the Netherlands, Ref. 162012), or the local ethical committee of the Leeds Teaching Hospitals NHS Trust (United Kingdom, Ref. 01/040). CFs were isolated as described above and cultured in DMEM (Gibco) supplemented with 10% FBS (Gibco), gentamicin (50 µg/mL, Gibco), 1% Insulin-Transferrin-Selenium-Sodium Pyruvate (ITS-A, Gibco), and basic fibroblast growth factor (1 ng/mL, Gibco). Cells were harvested, frozen and stored in liquid nitrogen, and used from passage 1–4.

### 2.3. Stimulation of CFs with Thrombin and FXa

Rat atrial or ventricular CFs were seeded at 10,000 cells/cm^2^, cultured for 24 h, and serum-starved for 24 h prior exposure to bovine thrombin (8 pM, Synapse, Maastricht, the Netherlands) or bovine FXa (100 nM, Synapse).

Similarly, human atrial CFs were seeded at 10,000 cells/cm^2^, cultured for 24 h, and serum-starved for 24 h, and subsequently exposed to purified human FXa (100 nM, Hyphen, Biomed, Neuville-sur-Oise, France) or PAR-1 agonist (TRAP-14, 100 µM, Bachem, Bubendorf, Switzerland). The selective FXa Inhibitor, rivaroxaban (400 ng/mL, Bayer, Leverkusen, Germany), was incubated with FXa for 10 min before addition to the cells. Effects on gene expression were determined at 4 and 24 h.

### 2.4. MCP-1 Assay

Conditioned media of rat CFs incubated with FXa for 24 h, were collected and stored at −80 °C for analysis. MCP-1 DuoSet ELISA was performed according to the manufacturer’s instructions (R&D Systems, Minneapolis, MN, USA).

### 2.5. Proliferation Assay

Human CFs (200 cells/cm^2^) were seeded in 96-well plates, cultured for 24 h, and serum-starved for 24 h. Subsequently, cells were incubated with human FXa (100 nM) with or without PAR-1 antagonist (1 µM, SCH79797, Tocris, Bioscience, Bristol, UK). All the stimuli were prepared in the presence of BrdU (10 µM) and 0.1% FBS. After 24 h of incubation with the stimuli, Cell Proliferation ELISA, BrdU (Roche, Basel, Switzerland) was performed according to the manufacturer’s instructions.

### 2.6. Gene Silencing

Adult human CFs were transfected with 10 nM *F2R* (PAR-1) or *F2RL1* (PAR-2), specific Silencer Select Pre-Designed siRNA (PAR-1: SASI-Hs01-00240436; PAR-2: SASI-Hs02-00339249, Merck) or MISSION SiRNA Universal Negative Control No. 1 siRNA (SCI001, Merck), using Lipofectamine RNAiMAX Reagent (Life Technologies, Carlsbad, CA, USA) in OptiMEM (Gibco), as per the manufacturer’s instructions. Medium was replaced with DMEM supplemented with 0.1% FBS 24 h later. Cells were used for experimentation 48 h after transfection.

### 2.7. Gene Expression Analysis

Total RNA was collected using the Micro-Elute Total RNA kit (Omega-Bio-Tek, Norcross, GA, USA) and reverse transcribed with Iscript into cDNA (Biorad, Hercules, CA, USA) according to the manufacturer’s protocol. Gene expression levels were measured with a CFX96 Touch Real-Time PCR detection system (BioRad) and SYBR Green Supermix technology (BioRad). Expression levels were normalized for Cyclophilin-A and calculated using the comparative threshold cycle method (ΔCt). The gene expression values were multiplied by 1000 to enhance readability. The sequences of the specific primers used are provided in Tables S1 and S2.

### 2.8. Statistical Analysis

All data in the figures are expressed as median bars, with *n* representing the number of cell isolations from different rats or patients, unless stated otherwise. Rat RT-qPCR analyses involving CTRL and either thrombin or FXa as the stimulus, and human RT-qPCR on *F2R* (PAR-1) and *F2RL1* (PAR-2), were statistically tested using the non-parametric Wilcoxon signed rank test. MCP-1 ELISA results were statistically analysed using the non-parametric Friedman test for paired samples and Dunn’s multiple comparison test. Human RT-qPCR including CTRL with FXa (or TRAP-14) and either siPAR-1 or siPAR-2 or rivaroxaban were tested for significance using the non-parametric Friedman test and Dunn’s multiple comparison test. The proliferation assay was statistically tested using a repeated-measures one-way ANOVA and Šidák’s post-hoc test. *p*-values < 0.05 were considered to be statistically significant. PRISM (version 9.0.0, GraphPad) was used to compute all statistics.

## 3. Results

### 3.1. Thrombin and FXa Induce Upregulation of Pro-Fibrotic and Proinflammatory Genes in Adult Rat CFs

**Figure 1:**
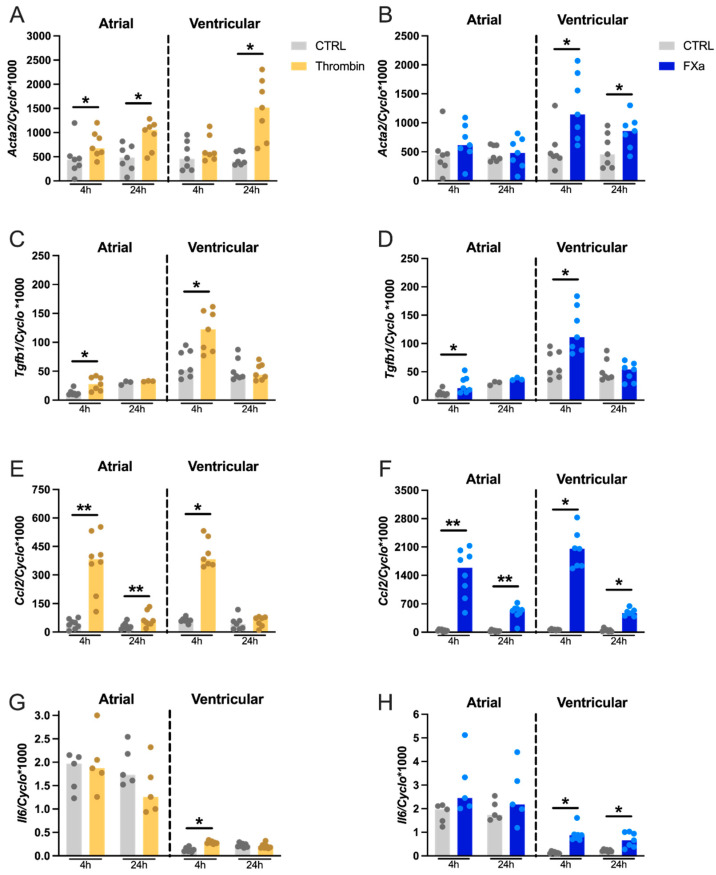
Effect of thrombin (**A**,**C**,**E**,**G**) and FXa (**B**,**D**,**F**,**H**) on mRNA expression of pro-fibrotic and proinflammatory genes. Rat atrial (*n* = 3–8) and ventricular (*n* = 7) CF were stimulated with 8 pM of thrombin or 100 nM of FXa for 4 and 24 h. Gene expression of *Acta2 (***A**,**B***), Tgfb1 (***C**,**D***), Ccl2 (***E**,**F***),* and *Il6 (***G**,**H***)* was measured by RT-qPCR. Data were normalized to the housekeeping gene Cyclophilin-A. Results are expressed as median bars, with dots indicating separate CF isolations. Statistical analysis was performed using the non-parametric Wilcoxon test for paired samples (* p < 0.05, ** p < 0.01).

Thrombin induced upregulation of *Acta2* mRNA at 4 and 24 h in atrial CFs (1.6-fold, 1.9-fold, respectively), while in ventricular CFs, this upregulation was significant only at 24 h (3.1-fold, Figure 1A). Exposure of ventricular CFs to FXa upregulated *Acta2* mRNA at both 4 and 24 h (1.6-fold and 2.7-fold, respectively, Figure 1B). No effect of FXa was observed on *Acta2* expression in atrial cells. Thrombin and FXa also upregulated the expression of *Tgfb1* (TGF-β1), another well-known pro-fibrotic marker, in both atrial and ventricular cells at 4 h (Thrombin: 2.2-fold and 1.9-fold, respectively, Figure 1C; FXa: 2.1-fold and 1.9-fold, respectively, Figure 1D).

**Figure 2:**
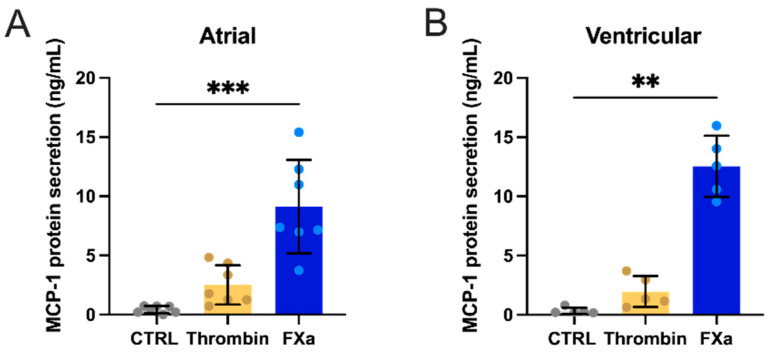
Effect of thrombin and FXa on MCP-1 protein secretion at 24 h in atrial CFs, *n* = 7 (**A**), and ventricular CFs, *n* = 5 (**B**). Statistical analysis was performed using the non-parametric Friedman test for paired samples (atrial: overall *p* < 0.0001; ventricular: overall *p* < 0.0001) and Dunn’s multiple comparison test (** *p* < 0.01, *** *p* < 0.001). Results are expressed as means ± SD.

Four-hour incubation of atrial or ventricular CFs with thrombin increased the gene expression of *Ccl2* (MCP-1) by 8.5-fold and 6.6-fold, respectively (Figure 1E). This induction was still significantly increased in atrial cells at 24 h (2.1-fold), although it was not as strong as at the earlier time point. In ventricular CF, *Ccl2* expression returned to baseline levels after 24 h. Interestingly, incubation of atrial and ventricular CF with FXa triggered fast and strong upregulation of *Ccl2* mRNA levels. After 4 h of incubation, *Ccl2* expression was increased by 34.2-fold in atrial and 32.4-fold in ventricular CFs. At 24 h, FXa was still able to induce overexpression of *Ccl2* by 16.1-fold in atrial and 10.4-fold-in ventricular CFs (Figure 1F). Furthermore, the strong induction of *Ccl2* expression mediated by FXa was reflected by the 20.6- (atrial) and 36.0-fold (ventricular) upregulation of MCP-1 protein levels in the conditioned media of CFs exposed to FXa for 24 h. Thrombin also increased secretion of MCP-1 although not as strongly (5.6-fold in both atrial and ventricular CFs, Figure 2). Moreover, thrombin upregulated the expression of another proinflammatory gene, *Il6* (IL-6), in ventricular CFs at 4 h (2.7-fold, Figure 1G). In atrial cells, the expression of *Il6* was not affected. These cells also showed a significantly higher baseline expression compared to ventricular CFs. *Il6* expression was also upregulated by FXa in ventricular cells at both time points (6.9-fold at 4 h and 3-fold at 24 h, Figure 1H). Atrial CF *Il6* mRNA levels showed a trend towards an increase at 4 h of FXa (*p* = 0.06).

**Figure 3:**
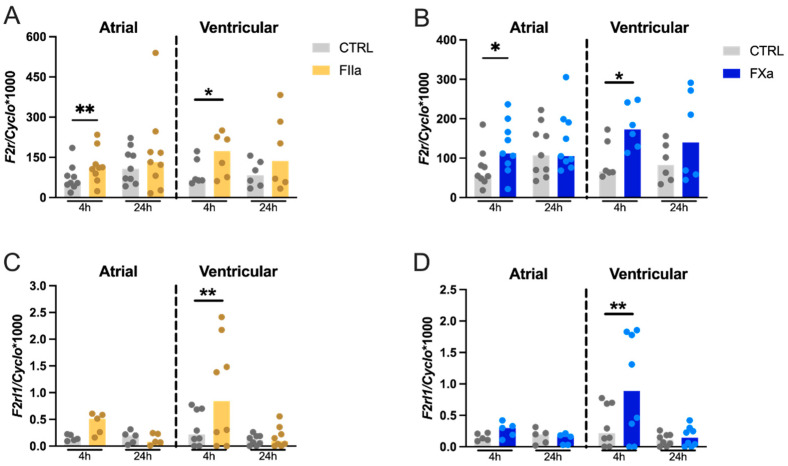
Effect of thrombin and FXa on mRNA expression of *F2r* (PAR-1 gene) (**A**,**B**) and *F2rl1* (PAR-2 gene) (**C**,**D**). Rat atrial (*n* = 5–9) and ventricular (*n* = 6–8) CFs were stimulated with 100 nM of FXa for 4 and 24 h. Gene expression of *F2r* and *F2rl1* was measured by RT-qPCR. Data were normalized to the housekeeping gene Cyclophilin-A. Results are expressed as median bars, with dots indicating individual rat CF isolations. Statistical analysis was performed using the non-parametric Wilcoxon test for paired samples (* *p* < 0.05, ** *p* < 0.01).

Finally, gene expression analysis revealed that adult rat CFs expressed PAR-1- and PAR-2-encoding genes, *F2r* and *F2rl1*, respectively (Figure 3), while mRNA levels of *F2rl2* and *F2rl3* (expressing the PAR-3 and PAR-4 proteins, respectively) were not detectable. Furthermore, rat CFs showed a much higher expression level of *F2r* compared to *F2rl1* mRNA, suggesting that PAR-1 is the most abundantly expressed PARs isoform in these cells. Exposure of atrial and ventricular cells to thrombin or FXa for 4 h significantly upregulated *F2r* gene expression (atrial:1.7-fold and 1.7-fold, respectively; ventricular: 1.7-fold and 1.9-fold, respectively, Figure 3A,B). Similarly, thrombin and FXa upregulated the gene expression of *F2rl1* in ventricular cells at 4 h (2.9-fold and 2.8-fold, respectively, Figure 3C,D), while no significant difference was found in atrial CFs.

### 3.2. Silencing of PAR-1 Attenuates FXa-Mediated Upregulation of CCL2 and IL6 in Primary Human Atrial CFs

**Figure 4:**
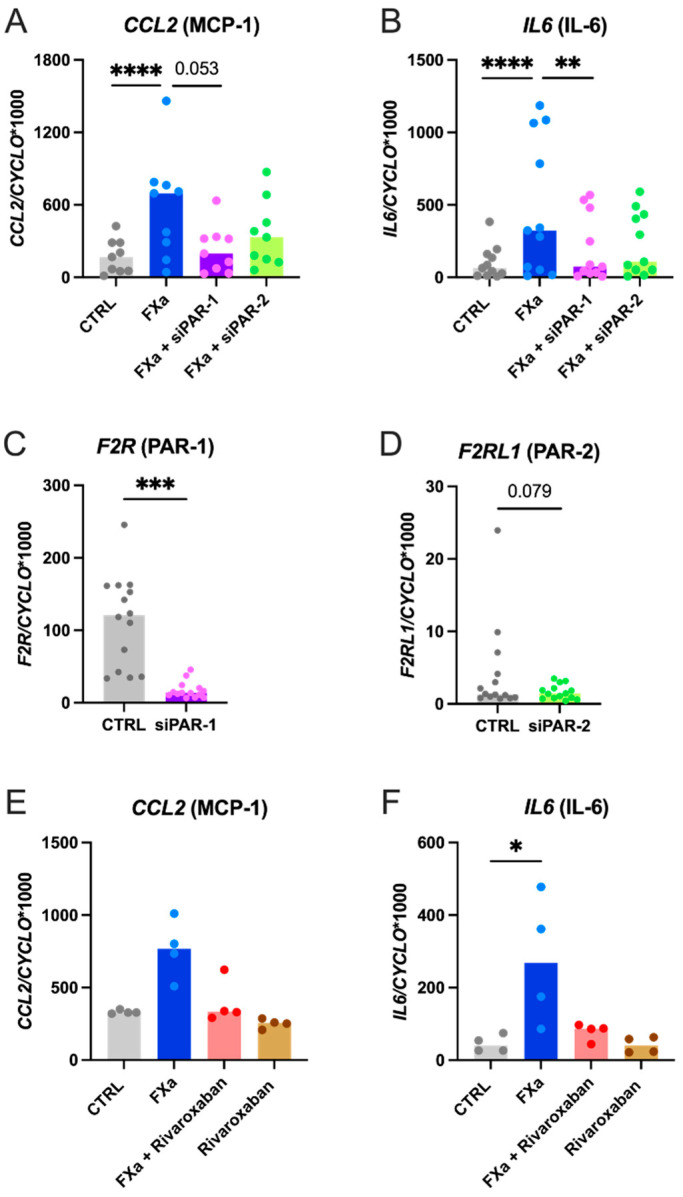
Effect of PARs silencing on FXa-mediated *CCL2* and *IL6* expression in human CFs (**A**,**B**; *n* = 8–9). Before exposure to FXa (100 nM), human atrial CFs were transfected with 10 μM of either scrambled *F2R* (PAR-1) or *F2RL1* (PAR-2) specific siRNA and incubated for 48 h. Gene expression was measured by RT-qPCR after 4 h of incubation with FXa. Data were normalized to the housekeeping gene Cyclophilin-A. Statistical analysis was performed using the non-parametric Friedman test for paired samples (*CCL2*: overall *p* < 0.001; *IL6*: overall *p* < 0.001) and Dunn’s multiple comparison test (** *p* < 0.01, **** *p* < 0.0001). Effect of silencing on *F2R* (PAR-1) and *F2Rl1* (PAR-2) gene expression (**C**,**D**; *n* = 14). Data were analysed using the non-parametric Wilcoxon test for paired samples (*** *p* < 0.001). Effect of FXa inhibition by rivaroxaban on *CCL2* and *IL6* expression in human CFs (**E**,**F**; *n* = 4). Statistical analysis was performed using the non-parametric Friedman test for paired samples (*CCL2:* overall *p* < 0.001; *IL6:* overall *p* < 0.001) and Dunn’s multiple comparison test (* *p* < 0.05). Results are expressed as median bars, with dots indicating individual human CF isolations.

The effect of FXa on the upregulation of *CCL2* and *IL6* gene expression was confirmed in primary human atrial CFs (3.4-fold and 4.6-fold, respectively, Figure 4A,B). Knockdown of the genes *F2R* or *F2RL1* (encoding PAR-1 and PAR-2 proteins) produced an 85% and 61% reduction of their relative expression, respectively (Figure 4C,D). Interestingly, silencing of *F2R* significantly reduced the effect of FXa on IL6 mRNA expression, while *F2RL1* knockdown did not significantly attenuate this effect (Figure 4A,B). Similarly, the FXa-induced increase in *CCL2* expression was lower upon *F2R* silencing, although this effect failed to reach statistical significance. Silencing of *F2RL1* seemed to downregulate the FXa-mediated *CCL2* increase but not as strongly as *F2R* knockdown (Figure 4A,B).

Furthermore, inhibition of FXa by its selective inhibitor rivaroxaban completely prevented the upregulation of *CCL2* and *IL6* (Figure 4E,F).

### 3.3. PAR-1 Agonist Induces Upregulation of CCL2 and IL6 in Human CFs

**Figure 5:**
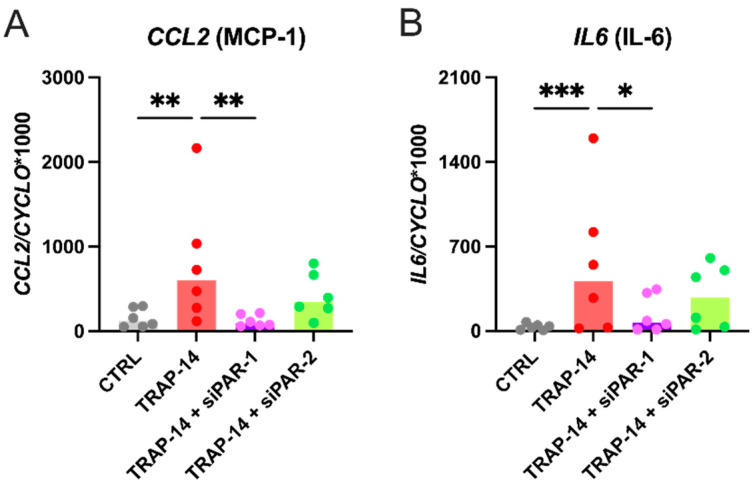
Effect of PARs silencing on *CCL2* and *IL6* expression (**A**,**B**; *n* = 6). Human atrial CFs were transfected with 10 μM of either scrambled PAR-1 or PAR-2 specific siRNA before exposure to TRAP-14 (100 μM). Gene expression was measured by RT-qPCR after 4 h of incubation. Data were normalized to the housekeeping gene Cyclophilin-A. Results are expressed as median bars, with dots indicating individual human CF isolations. Statistical analysis was performed using the non-parametric Friedman test (*CCL2*: overall *p* < 0.0001; *IL6*: overall *p* < 0.0001) test for paired samples and Dunn’s multiple comparison test (* *p <* 0.05, ** *p* < 0.01, *** *p* < 0.001).

PAR-1 activation by TRAP-14, a selective PAR-1 agonist, significantly upregulated the gene expression of *CCL2* (5.1-fold) and *IL6* (15.9-fold) mRNAs in human atrial CFs at 4 h, respectively (Figure 5A,B). As expected, PAR-1 silencing fully prevented this effect while PAR-2 knockdown did not.

### 3.4. PAR-1 Antagonist Downregulates the Effect of FXa on Human CF Proliferation

**Figure 6:**
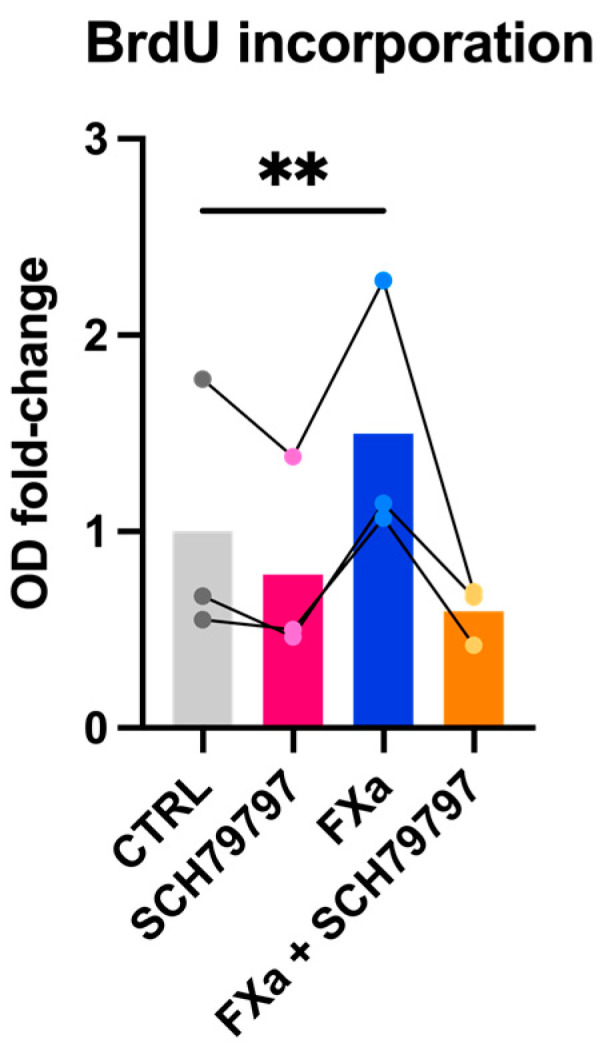
Proliferation assay on human atrial CFs (*n* = 3). Cells were incubated with 100 nM FXa with and without SCH79797 (1 μM). Proliferation was measured 24 h after exposure to the stimuli via BrdU colorimetric assay. Results are expressed as fold-change relative to CTRL (mean bars). Statistical analysis was performed using the repeated measures one-way ANOVA (*p* < 0.01) and Šidák’s multiple comparison test (** *p* < 0.01). Lines depict paired data points.

Exposure of human CFs to FXa for 24 h stimulated cell proliferation compared to control-treated cells (1.5-fold). This proliferative effect of FXa was reduced by a selective PAR-1 antagonist (SCH79797), although this reduction failed to reach statistical significance (Figure 6).

## 4. Discussion

This study shows that FXa upregulates the gene expression of two key regulators of inflammatory processes, *CCL2* and *IL6*, in primary adult human atrial CFs. Surprisingly, the FXa pro-inflammatory effect was mainly mediated by PAR-1 activation, which appears to be the most abundant isoform expressed in CFs. PAR-1 agonist also increases *CCL2* and *IL6* gene expression, confirming that PAR-1 activation plays a pivotal role in inflammatory processes mediated by CFs.

Moreover, our data show that the coagulation factors thrombin and FXa lead to increased expression of well-known pro-fibrotic genes in primary adult rat CFs, supporting the possible link between (pathological) activation of the coagulation system and cardiac remodelling.

### 4.1. Coagulation Factors Induce Pro-Fibrotic and Proinflammatory Gene Expression Changes in Rat CFs

In this study we made use of primary adult rat CFs to explore the effect of thrombin and FXa on CF activation. In line with what is reported in the literature [7], thrombin induced overexpression of the *Acta2* and *Tgfb1* genes in atrial CFs already after 4 h. In a similar fashion, FXa upregulated the expression of these pro-fibrotic markers mainly in ventricular CFs. This suggests that both thrombin and FXa may play a role in the activation of CFs within the process of cardiac remodelling.

PARs are the main mediators of cellular signalling elicited by coagulation factors including thrombin and FXa [9]. To elucidate their role in this process, we tested whether PAR activation would also lead to upregulation of their mRNA levels. Interestingly, both thrombin and FXa were able to upregulate the expression of the rat *F2r* and *F2rl1* genes, encoding PAR-1 and PAR-2, respectively, in CFs. These results are in line with what has previously been described by our group and suggest the existence of a positive feedback loop on PAR expression upon their activation [7].

Most importantly, our study confirmed the proinflammatory effect of FXa and provided additional insight on its cellular signalling on CFs. As described by several in vitro and in vivo studies, we found that exposure of rat CFs to FXa resulted in a strong and rapid upregulation of *Ccl2* (in both atrial and ventricular CFs) and *IL6* (in ventricular CFs) mRNA levels [11,12]. Moreover, *Ccl2* overexpression was accompanied by increased MCP-1 protein levels, detected in the conditioned media of CFs exposed to FXa for 24 h.

### 4.2. Coagulation Factors Elicit Inflammatory Signalling through PAR-1 in Human CFs

FXa-mediated proinflammatory changes were confirmed in primary adult human atrial CFs, where the expression of *CCL2* and *IL6* increased upon stimulation with FXa for 4 h.

To investigate the mechanism by which FXa leads to inflammatory responses in human CFs, *F2R* (PAR-1) and *F2RL1* (PAR-2) were silenced. Surprisingly, *F2RL1* silencing did not significantly alter the FXa-mediated upregulation of *CCL2* and *IL6.* In contrast, our experiments revealed that *F2R* silencing did prevent the upregulation of *CCL2* and *IL6* by FXa. These results suggest that FXa elicits its proinflammatory role mainly via PAR-1 activation. Moreover, in line with what was reported by Snead and colleagues, at baseline, human (and rat) CFs expressed much higher *F2R* than *F2RL1* mRNAs, indicating that PAR-1 might be the predominant isoform in these cells [17].

Our findings seem to disagree with the literature about the role of PAR-2 as the main mediator of FXa proinflammatory activity [9,18]. For example, Bukowska and colleagues reported that incubation of human atrial tissue slices with FXa increased mRNA expression of inflammatory molecules, such as intercellular adhesion molecules and IL-8. These effects were mediated by PAR-2 activation and downregulated by the direct FXa inhibitor, rivaroxaban [19]. A possible explanation to the seemingly contrasting data between our study and previous reports is the fact that the cardiac tissue slices contained all cardiac cell types, while the specific effect of FXa on CFs was not characterized. Recently, the same research group showed that exposure of A549 cells (model of type two alveolar epithelial cells) to FXa upregulates the gene expression of inflammatory molecules, such as MCP-1. Interestingly, in these cells, the effect of FXa on proinflammatory markers was prevented by PAR-1 inhibition [20]. These data support our findings and suggest that FXa proinflammatory signalling via PAR-1 may also play a relevant role in other organs and cell types.

Moreover, our data show that, in human atrial CFs, rivaroxaban downregulated the effect of FXa on *IL6* and *CCL2* expression. These results indicate that FXa is the actual mediator of these proinflammatory changes and exclude the possible contribution of thrombin (of which small traces may be present in FXa preparation) in the upregulation of these markers.

Furthermore, in line with what was previously described by Guo et al. in neonatal rat CFs, we showed that FXa enhanced the proliferation of human CFs and that this effect seemed to be attenuated by PAR-1 antagonist [21].

Finally, to further elucidate the role of PAR-1 signalling, CFs were incubated with a selective PAR-1 agonist that significantly upregulated the gene expression of *CCL2* and *IL6* after 4 h. As expected, the upregulation of these genes was fully prevented by PAR-1 knockdown but not by PAR-2 knockdown. Interestingly, upon PAR-2 silencing, PAR-1 agonist was not able to induce an upregulation as strong as PAR-1 agonist alone, suggesting that PAR-2 might still play a role in the gene expression of these markers possibly via its transactivation by PAR-1 [22].

Taken together, these data confirm that PAR-1 activation triggers early upregulation of proinflammatory genes in human CFs and therefore plays a pivotal role in inflammatory responses mediated by CFs.

## 5. Study Limitations

In the present study, primary adult human atrial CFs were used to investigate the signalling receptor involved in the proinflammatory effect of FXa found in rat CFs. As extensively reported in the literature, prolonged in vitro culturing of CFs promotes their activation and differentiation into myofibroblasts. To preserve the CF phenotype, our experiments were performed on cells at a low passage number (the highest passage number was 4). However, this, in combination with the low proliferation rate and the relatively small number of donors, limited the number of experiments (and conditions) we could perform. Nevertheless, we were able to carry out key experiments and answer our primary research questions.

Finally, we reported the effect of FXa on the upregulation of only two proinflammatory genes, *CCL2* and *IL6.* Despite the fact that these markers are two well-known regulators of proinflammatory processes, further analysis on other regulators and targets can be helpful to clarify the mechanism of action of FXa signalling on CFs. Moreover, co-culture experiments and migration assays on CFs in combination with immune cells can be performed to further characterize the proinflammatory character of FXa and study the functionality of inflammatory changes in CFs.

## 6. Conclusions

This study demonstrates that FXa induces proinflammatory responses in human atrial CFs by upregulating the expression of two well-known regulators of inflammatory processes, *CCL2* and *IL6*. Moreover, our data show that FXa elicits cellular signalling mainly via activation of PAR-1, which was found to be the most abundantly expressed PARs isoform in primary adult human (and rat) CFs.

## Data Availability

The data presented in this study are available from the corresponding author upon request.

## Supplementary Materials

The following are available online at https://www.mdpi.com/article/10.3390/cells10112958/s1, Table S1: Gene-specific Rattus norvegicus primer sequences used for quantitative real-time PCR, Table S2: Gene-specific Homo sapiens primer sequences used for quantitative real-time PCR.
