# Supplementary material for: Coagulation Factor Xa Induces Proinflammatory Responses in Cardiac Fibroblasts via Activation of Protease-Activated Receptor-1
Source: Cells. 2021 Oct 30;10(11):2958. doi: 10.3390/cells10112958 (PMC8616524; doi:10.3390/cells10112958)
Supplement: Supplementary file 1 [file cells-10-02958-s001.zip › cells-1414620-supplementary.pdf]

Coagulation Factor Xa Induces Proinflammatory Responses in Cardiac Fibroblasts via Activation of Protease-Activated Receptor-1

Elisa D'Alessandro [1], Billy Scaf [2], Chantal Munts [2], Arne van Hunnik [2], Christopher J. Trevelyan [3,4], Sander Verheule [2], Henri M. H. Spronk [1], Neil A. Turner [3,4], Hugo ten Cate [1,5], Ulrich Schotten[2] and Frans A. van Nieuwenhoven [2]

[1] Departments of Biochemistry and Internal medicine, Cardiovascular Research Institute Maastricht, Maastricht University Medical Center, Maastricht, Netherlands
[2] Department of Physiology, Cardiovascular Research Institute Maastricht, Maastricht University Medical Center, Maastricht, Netherlands
[3] Discovery and Translational Science Department, Leeds Institute of Cardiovascular and Metabolic Medicine, School of Medicine, University of Leeds, UK
[4] Multidisciplinary Cardiovascular Research Centre, University of Leeds, UK
[5] Center for Thrombosis and Haemostasis, Gutenberg University Medical Centre, Mainz, Germany
* Correspondence: f.vannieuwenhoven@maastrichtuniversity.nl

Supplemental Table S1. Gene-specific Rattus norvegicus primer sequences used for quantitative real-time PCR

Target gene	Symbol	Forward primer	Reverse primer
Alpha-smooth muscle actin	*(Acta2)*	AAGGCCAACCGGGAGAAAAT	AGTCCAGCACAATACCAGTTGT
C-C motif chemokine ligand 2	*(Ccl2)*	GCCTGTTGTTCACAGTTGCT	AGTTCTCCAGCCGACTCATT
Coagulation factor II (thrombin) receptor	*(F2r)*	GCCAGAAGCACCTTTACAGC	TTCAGGTGGCTAGAGCAGGT
F2R like trypsin receptor 1	*(F2rl1)*	GAGGTATCACCCTTCTGGCG	GCGTGTCCAATCTGCCAATC
Interleukin 6	*(Il6)*	GATACCACCCACAACAGACC	CATTTCCAAGATCTCCCTGA
Transforming growth factor, beta 1	*(Tgfb1)*	GCACCATCCATGACATGAAC	GCTGAAGCAGTAGTTGGTATC
Cyclophilin-A	*(Cyclo)*	CAAATGCTGGACCAAACACAA	TTCACCTTCCCAAAGACCACAT

Supplemental Table S2. Gene-specific Homo sapiens primer sequences used for quantitative real-time PCR

Target gene	Symbol	Forward primer	Reverse primer
Alpha-smooth muscle actin	*(ACTA2)*	TGTGCTGGACTCTGGAGATG	GAAGGAATAGCCACGCTCAG
C-C motif chemokine ligand 2	*(CCL2)*	CATGAAAGTCTCTGCCGCCC	GGGCATTGATTGCATCTGG
Coagulation factor II (thrombin) receptor	*(F2R)*	CCGCAGGCCAGAATCAAAAG	TTCTCCTCATCCTCCCAAAATGG
F2R like trypsin receptor 1	*(F2RL1)*	GCTCTGAGTTTCGAATCGGC	GTTCCTTGGATGGTGCCACT
Interleukin 6	*(IL6)*	AGTCCTGATCCAGTTCCTGC	CTGGCATTTGTGGTTGGGTC
Transforming growth factor, beta 1	*(TGFB1)*	CGACTCGCCAGAGTGGTTAT	GTGAACCCGTTGATGTCCA
Cyclophilin-A	*(CYCLO)*	CCCACCGTGTTCTTCGACAT	CCAGTGCTCAGAGCACGAAA
